# 4-(Dodec­yloxy)benzonitrile

**DOI:** 10.1107/S1600536811041602

**Published:** 2011-10-22

**Authors:** Huey Chong Kwong, Mohamad Zaki Ab. Rahman, Mohamed Ibrahim Mohamed Tahir, Sidik Silong

**Affiliations:** a Department of Chemistry, Faculty of Science, Universiti Putra Malaysia, 43400 UPM Serdang, Selangor, Malaysia

## Abstract

In the title compound, C_19_H_29_NO, the C—C and C—N bond distances of the benzonitrile group are 1.445 (2) and 1.157 (2) Å, respectively. The aliphatic fragment adopts a bent zigzag arangement which differs from the planar zigzag arrangement normally observed in *n*-alkanes or long-chain alkyl­benzenes. In the crystal, inversion dimers linked by pairs of C—H⋯O hydrogen bonds occur. A C—H⋯N inter­action also occurs. In the crystal, mol­ecules are packed with the nitrile and aliphatic groups oriented in a head-to-tail fashion involving, forming a ripple-like motif along the *a* axis.

## Related literature

For standard bond lengths, see Allen *et al.* (1987 ▶). For related structures, see: Merz (2002 ▶); Britton *et al.* (2004 ▶); Kwong *et al.* (2011 ▶); Boese *et al.* (1999 ▶). The title compound was synthesised by reacting hy­droxy­benzonitrile with bromo­alkane, see Rahman *et al.* (2009 ▶).
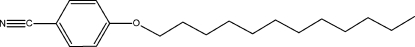


## Experimental

### 

#### Crystal data

C_19_H_29_NO
*M* *_r_* = 287.45
Monoclinic, 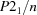

*a* = 5.7080 (6) Å
*b* = 7.3644 (8) Å
*c* = 40.642 (5) Å
β = 90°
*V* = 1708.4 (3) Å^3^
*Z* = 4
Cu *K*α radiation
μ = 0.52 mm^−1^
*T* = 100 K
0.17 × 0.14 × 0.09 mm

#### Data collection

Oxford Diffraction Gemini E diffractometer
Absorption correction: multi-scan (*CrysAlis PRO*; Oxford Diffraction, 2006 ▶) *T*_min_ = 0.930, *T*_max_ = 0.955
9444 measured reflections
3214 independent reflections
2575 reflections with *I* > 2σ(*I*)
*R*_int_ = 0.020

#### Refinement

*R*[*F* ^2^ > 2σ(*F* ^2^)] = 0.046
*wR*(*F* ^2^) = 0.127
*S* = 0.98
3202 reflections
190 parameters
H-atom parameters constrained
Δρ_max_ = 0.24 e Å^−3^
Δρ_min_ = −0.25 e Å^−3^

### 

Data collection: *Gemini* (Oxford Diffraction, 2006 ▶); cell refinement: *CrysAlis RED* (Oxford Diffraction, 2006 ▶); data reduction: *CrysAlis RED*; program(s) used to solve structure: *SIR92* (Altomare *et al.*, 1994 ▶); program(s) used to refine structure: *CRYSTALS* (Betteridge *et al.*, 2003 ▶); molecular graphics: *Mercury* (Macrae *et al.*, 2006 ▶); software used to prepare material for publication: *CRYSTALS*.

## Supplementary Material

Crystal structure: contains datablock(s) global, I. DOI: 10.1107/S1600536811041602/kp2353sup1.cif

Structure factors: contains datablock(s) I. DOI: 10.1107/S1600536811041602/kp2353Isup2.hkl

Supplementary material file. DOI: 10.1107/S1600536811041602/kp2353Isup3.cml

Additional supplementary materials:  crystallographic information; 3D view; checkCIF report

## Figures and Tables

**Table 1:** Hydrogen-bond geometry (Å, °)

*D*—H⋯*A*	*D*—H	H⋯*A*	*D*⋯*A*	*D*—H⋯*A*
C10—H102⋯N7^i^	0.98	2.67	3.468 (2)	139
C3—H31⋯O1^ii^	0.94	2.67	3.569 (5)	159

Symmetry codes: (i) 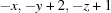; (ii) 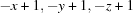.

**Fig. 1.**
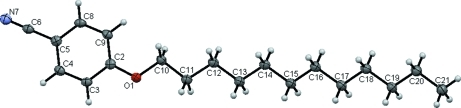
Molecular structure of (I) with atom numbering and displacement ellipsoids at 50% probability level.

**Fig. 2.**
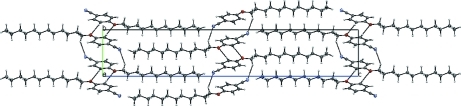
The packing diagram of (I) showing a ripple-like motif viewing along a axis; hydrogen bonds were shown as dashed lines [b axis green; c axis blue].

## supplementary crystallographic information

### Comment

The titled compound (I), 4-(dodecyloxy)benzonitrile (Fig. 1) was synthesised by reacting hydroxybenzonitrile with bromoalkane (Rahman *et al.*, 2009). Bond distance and angles of (I) are in normal range (Allen *et al.* 1987). Bond distance of the benzonitrile group C5—C6 and C6—N7 are 1.445 (2) Å and 1.157 (2) Å, respectively and these bond lengths are comparable with those in *p*-decylbenzonitrile of 1.446 (3) Å and 1.153 (3) Å, respectively (Britton *et al.*, 2004).

In this molecule, the plane formed by benzonitrile ring and O1 was almost planar, the largest deviation from the least-squares plane is 0.0187 (12) Å at O1. The benzene ring and the alkane carbon skeleton (C9—C2—O1—C10) form the torsion angle of 1.62 (2)°. In this structure the alkane carbon skeleton has a bended zigzag arrangement; this arrangement is in agreement with previously reported alkoxy benzenen [4-hexyloxybenzamide, Kwong *et al.*, 2011] However, the mean C(H3)—C(H2) and C(H2)—C(H2) distances, and C(H3)—C(H2)—C and C(H2)—C(H2)—C angles, are in accordance of those determined for n-alkanes and long-chain alkylbenzene, 1.521 (1) Å and 112.8 (1)–113.5 (1)°, respectively. (Boese *et al.*, 1999; Merz, 2002; Britton *et al.*, 2004).

In the crystal packing, the centrosymmetric hydrogen bond C3—H31···O1 is formed generating a hydrogen bonded ring (Table 1 and Fig. 2). Packing of the titled compound shows a ripple-like motif (Fig. 2) with nitrile and aliphatic groups oriented head-to-tail. The stacking interaction between the aromatic rings with the separation distances of their centres of gravity Cg1···Cg1^i^ (-x,1-y,1-z) of 3.573 (1) and Cg1···Cg1^ii^(-x,2-y,1-z) of 3.808 (1) Å and slipage of 1.395 and 1.865 Å, respectively, were observed.

### Experimental

The titled compound (I) was synthesised by reacting hydroxybenzonitrile with bromoalkane with conventional heating (Rahman *et al.*, 2009). Crystals of (I) were grown from hexane using a slow evaporation.

### Refinement

The H atoms were all located in a difference map, but those attached to carbon atoms were repositioned geometrically. The H atoms were initially refined with soft restraints on the bond lengths and angles to regularize their geometry (C—H in the range 0.93–0.98, N—H in the range 0.86–0.90 Å) and *U*_iso_(H) (in the range 1.2–1.5 times *U*_eq_ of the parent atom), after which the positions were refined with riding constraints.

### Crystal data

**Table:**

C_19_H_29_NO	*F*(000) = 632
*M**_r_* = 287.45	*D*_x_ = 1.117 Mg m^−^^3^
Monoclinic, *P*2_1_/*n*	Cu *K*α radiation, λ = 1.54180 Å
*a* = 5.7080 (6) Å	Cell parameters from 3853 reflections
*b* = 7.3644 (8) Å	θ = 3–71°
*c* = 40.642 (5) Å	µ = 0.52 mm^−^^1^
β = 90°	*T* = 100 K
*V* = 1708.4 (3) Å^3^	Plate-like, colourless
*Z* = 4	0.17 × 0.14 × 0.09 mm

### Data collection

**Table:**

Oxford Diffraction Gemini E diffractometer	3214 independent reflections
Radiation source: sealed x-ray tube	2575 reflections with *I* > 2σ(*I*)
graphite	*R*_int_ = 0.020
ω/2θ scans	θ_max_ = 71.6°, θ_min_ = 4.4°
Absorption correction: multi-scan (*CrysAlis PRO*; Oxford Diffraction, 2006)	*h* = −6→6
*T*_min_ = 0.930, *T*_max_ = 0.955	*k* = −8→9
9444 measured reflections	*l* = −37→49

### Refinement

**Table:**

Refinement on *F*^2^	Primary atom site location: structure-invariant direct methods
Least-squares matrix: full	Hydrogen site location: difference Fourier map
*R*[*F*^2^ > 2σ(*F*^2^)] = 0.046	H-atom parameters constrained
*wR*(*F*^2^) = 0.127	Method = Modified Sheldrick *w* = 1/[σ^2^(*F*^2^) + (0.05*P*)^2^ + 1.35*P*], where *P* = [max(*F*_o_^2^,0) + 2*F*_c_^2^]/3
*S* = 0.98	(Δ/σ)_max_ = 0.007
3202 reflections	Δρ_max_ = 0.24 e Å^−^^3^
190 parameters	Δρ_min_ = −0.25 e Å^−^^3^
0 restraints	

### Special details

**Table:**

Refinement. Refinement. For this compound, 9444 numbers of reflections were collected and measured during the refinement. Symmetry related reflections were measured more than once and after merging the symmetry equivalent reflections there were only 3214 reflection left. 12 more reflections were filtered, as σ cutoff was set as 3 and (sin?/*x*)set to>0.01 (to eliminate reflection measured near the vicinity of beam stop) therefore numbers of reflection reduced to 3202.

### Fractional atomic coordinates and isotropic or equivalent isotropic displacement parameters (Å^2^)

**Table:**

	*x*	*y*	*z*	*U*_iso_*/*U*_eq_	
O1	0.33735 (19)	0.61108 (16)	0.45325 (3)	0.0243	
C2	0.1734 (3)	0.6793 (2)	0.47454 (4)	0.0214	
C3	0.2277 (3)	0.6631 (2)	0.50786 (4)	0.0221	
C4	0.0719 (3)	0.7247 (2)	0.53150 (4)	0.0228	
C5	−0.1403 (3)	0.8033 (2)	0.52206 (4)	0.0219	
C6	−0.3025 (3)	0.8685 (2)	0.54668 (4)	0.0234	
N7	−0.4333 (3)	0.9209 (2)	0.56623 (4)	0.0295	
C8	−0.1922 (3)	0.8209 (2)	0.48877 (4)	0.0221	
C9	−0.0351 (3)	0.7606 (2)	0.46502 (4)	0.0220	
C10	0.2944 (3)	0.6284 (2)	0.41859 (4)	0.0242	
C11	0.5019 (3)	0.5506 (2)	0.40024 (4)	0.0252	
C12	0.4902 (3)	0.5918 (2)	0.36351 (4)	0.0246	
C13	0.6978 (3)	0.5162 (2)	0.34403 (4)	0.0257	
C14	0.7033 (3)	0.5790 (2)	0.30831 (4)	0.0251	
C15	0.9124 (3)	0.5066 (2)	0.28868 (4)	0.0258	
C16	0.9230 (3)	0.5776 (2)	0.25349 (4)	0.0257	
C17	1.1323 (3)	0.5068 (2)	0.23378 (4)	0.0257	
C18	1.1450 (3)	0.5806 (2)	0.19882 (4)	0.0258	
C19	1.3543 (3)	0.5106 (2)	0.17902 (4)	0.0259	
C20	1.3684 (3)	0.5847 (3)	0.14407 (4)	0.0282	
C21	1.5784 (3)	0.5133 (3)	0.12487 (4)	0.0322	
H31	0.3704	0.6093	0.5144	0.0260*	
H41	0.1098	0.7150	0.5546	0.0259*	
H81	−0.3353	0.8725	0.4821	0.0251*	
H91	−0.0710	0.7743	0.4422	0.0250*	
H102	0.2751	0.7577	0.4131	0.0287*	
H101	0.1498	0.5632	0.4128	0.0284*	
H111	0.6426	0.6050	0.4091	0.0296*	
H112	0.5060	0.4186	0.4040	0.0302*	
H122	0.4875	0.7226	0.3605	0.0291*	
H121	0.3473	0.5416	0.3544	0.0290*	
H131	0.8392	0.5561	0.3546	0.0305*	
H132	0.6919	0.3834	0.3445	0.0313*	
H142	0.7085	0.7111	0.3080	0.0303*	
H141	0.5615	0.5402	0.2974	0.0293*	
H151	1.0537	0.5428	0.3000	0.0304*	
H152	0.9037	0.3730	0.2881	0.0309*	
H162	0.9305	0.7093	0.2541	0.0313*	
H161	0.7809	0.5426	0.2420	0.0302*	
H172	1.2745	0.5408	0.2450	0.0312*	
H171	1.1235	0.3737	0.2328	0.0314*	
H181	1.1543	0.7134	0.1998	0.0308*	
H182	1.0027	0.5487	0.1872	0.0310*	
H192	1.4972	0.5440	0.1907	0.0307*	
H191	1.3442	0.3777	0.1780	0.0315*	
H201	1.3788	0.7175	0.1451	0.0338*	
H202	1.2254	0.5535	0.1326	0.0335*	
H212	1.5813	0.5587	0.1021	0.0465*	
H211	1.7246	0.5486	0.1352	0.0467*	
H213	1.5746	0.3804	0.1239	0.0474*	

### Atomic displacement parameters (Å^2^)

**Table:**

	*U*^11^	*U*^22^	*U*^33^	*U*^12^	*U*^13^	*U*^23^
O1	0.0228 (6)	0.0292 (7)	0.0208 (6)	0.0029 (5)	−0.0019 (5)	−0.0005 (5)
C2	0.0216 (8)	0.0179 (8)	0.0247 (8)	−0.0037 (7)	−0.0010 (6)	−0.0006 (7)
C3	0.0195 (8)	0.0198 (8)	0.0269 (9)	−0.0008 (7)	−0.0045 (6)	0.0010 (7)
C4	0.0238 (9)	0.0212 (9)	0.0235 (8)	−0.0035 (7)	−0.0044 (7)	0.0001 (7)
C5	0.0208 (8)	0.0199 (8)	0.0251 (8)	−0.0043 (7)	0.0002 (6)	0.0002 (7)
C6	0.0224 (9)	0.0215 (8)	0.0261 (9)	−0.0022 (7)	−0.0055 (7)	0.0019 (7)
N7	0.0269 (8)	0.0338 (9)	0.0279 (8)	0.0001 (7)	−0.0017 (6)	−0.0015 (7)
C8	0.0173 (8)	0.0207 (9)	0.0283 (9)	−0.0022 (6)	−0.0044 (6)	0.0016 (7)
C9	0.0223 (8)	0.0211 (9)	0.0224 (8)	−0.0037 (7)	−0.0039 (6)	0.0016 (6)
C10	0.0231 (9)	0.0274 (9)	0.0221 (8)	−0.0011 (7)	−0.0031 (7)	0.0000 (7)
C11	0.0227 (9)	0.0283 (9)	0.0247 (9)	0.0004 (7)	−0.0018 (7)	−0.0009 (7)
C12	0.0226 (8)	0.0264 (9)	0.0249 (9)	−0.0001 (7)	−0.0024 (7)	0.0007 (7)
C13	0.0257 (9)	0.0274 (9)	0.0240 (9)	0.0034 (7)	−0.0025 (7)	−0.0012 (7)
C14	0.0228 (9)	0.0282 (9)	0.0244 (9)	0.0010 (7)	−0.0026 (7)	−0.0001 (7)
C15	0.0249 (9)	0.0277 (9)	0.0249 (9)	0.0026 (7)	−0.0036 (7)	−0.0016 (7)
C16	0.0251 (9)	0.0277 (9)	0.0242 (9)	0.0011 (7)	−0.0032 (7)	0.0001 (7)
C17	0.0254 (9)	0.0283 (9)	0.0234 (9)	0.0010 (7)	−0.0041 (7)	−0.0013 (7)
C18	0.0239 (9)	0.0284 (9)	0.0251 (9)	0.0009 (7)	−0.0034 (7)	0.0008 (7)
C19	0.0255 (9)	0.0281 (9)	0.0240 (9)	0.0003 (7)	−0.0040 (7)	−0.0016 (7)
C20	0.0270 (9)	0.0313 (10)	0.0264 (9)	0.0000 (8)	−0.0026 (7)	0.0006 (7)
C21	0.0308 (10)	0.0387 (11)	0.0269 (9)	−0.0008 (8)	−0.0012 (8)	−0.0007 (8)

### Geometric parameters (Å, °)

**Table:**

O1—C2	1.3698 (19)	C13—H132	0.979
O1—C10	1.4355 (19)	C14—C15	1.531 (2)
C2—C3	1.394 (2)	C14—H142	0.973
C2—C9	1.387 (2)	C14—H141	0.966
C3—C4	1.385 (2)	C15—C16	1.524 (2)
C3—H31	0.943	C15—H151	0.967
C4—C5	1.396 (2)	C15—H152	0.985
C4—H41	0.965	C16—C17	1.530 (2)
C5—C6	1.445 (2)	C16—H162	0.972
C5—C8	1.391 (2)	C16—H161	0.970
C6—N7	1.157 (2)	C17—C18	1.523 (2)
C8—C9	1.390 (2)	C17—H172	0.963
C8—H81	0.941	C17—H171	0.982
C9—H91	0.954	C18—C19	1.530 (2)
C10—C11	1.512 (2)	C18—H181	0.980
C10—H102	0.984	C18—H182	0.968
C10—H101	0.984	C19—C20	1.524 (2)
C11—C12	1.525 (2)	C19—H192	0.976
C11—H111	0.967	C19—H191	0.981
C11—H112	0.984	C20—C21	1.524 (2)
C12—C13	1.530 (2)	C20—H201	0.981
C12—H122	0.971	C20—H202	0.967
C12—H121	0.969	C21—H212	0.985
C13—C14	1.524 (2)	C21—H211	0.969
C13—H131	0.961	C21—H213	0.980
			
C2—O1—C10	118.08 (12)	C15—C14—H142	108.6
O1—C2—C3	115.50 (14)	C13—C14—H141	109.3
O1—C2—C9	124.62 (14)	C15—C14—H141	108.1
C3—C2—C9	119.89 (15)	H142—C14—H141	108.4
C2—C3—C4	120.20 (15)	C14—C15—C16	113.61 (14)
C2—C3—H31	120.0	C14—C15—H151	107.8
C4—C3—H31	119.8	C16—C15—H151	108.7
C3—C4—C5	120.14 (15)	C14—C15—H152	108.7
C3—C4—H41	120.4	C16—C15—H152	108.8
C5—C4—H41	119.4	H151—C15—H152	109.2
C4—C5—C6	120.23 (15)	C15—C16—C17	113.93 (14)
C4—C5—C8	119.37 (15)	C15—C16—H162	108.6
C6—C5—C8	120.40 (15)	C17—C16—H162	108.6
C5—C6—N7	179.56 (17)	C15—C16—H161	109.0
C5—C8—C9	120.54 (15)	C17—C16—H161	108.1
C5—C8—H81	120.1	H162—C16—H161	108.3
C9—C8—H81	119.4	C16—C17—C18	113.81 (14)
C8—C9—C2	119.85 (15)	C16—C17—H172	108.8
C8—C9—H91	120.1	C18—C17—H172	107.9
C2—C9—H91	120.0	C16—C17—H171	108.7
O1—C10—C11	108.46 (13)	C18—C17—H171	108.8
O1—C10—H102	109.2	H172—C17—H171	108.7
C11—C10—H102	110.0	C17—C18—C19	114.02 (14)
O1—C10—H101	109.5	C17—C18—H181	108.6
C11—C10—H101	110.8	C19—C18—H181	108.4
H102—C10—H101	108.9	C17—C18—H182	109.1
C10—C11—C12	111.90 (14)	C19—C18—H182	108.5
C10—C11—H111	108.0	H181—C18—H182	108.0
C12—C11—H111	108.6	C18—C19—C20	114.25 (14)
C10—C11—H112	108.4	C18—C19—H192	108.1
C12—C11—H112	110.4	C20—C19—H192	108.7
H111—C11—H112	109.4	C18—C19—H191	108.2
C11—C12—C13	113.60 (14)	C20—C19—H191	108.8
C11—C12—H122	108.7	H192—C19—H191	108.7
C13—C12—H122	108.0	C19—C20—C21	113.30 (15)
C11—C12—H121	109.5	C19—C20—H201	108.7
C13—C12—H121	108.5	C21—C20—H201	108.5
H122—C12—H121	108.5	C19—C20—H202	108.7
C12—C13—C14	113.50 (14)	C21—C20—H202	109.5
C12—C13—H131	107.9	H201—C20—H202	108.0
C14—C13—H131	108.5	C20—C21—H212	112.2
C12—C13—H132	109.1	C20—C21—H211	111.4
C14—C13—H132	108.8	H212—C21—H211	107.5
H131—C13—H132	109.0	C20—C21—H213	110.4
C13—C14—C15	114.01 (14)	H212—C21—H213	107.5
C13—C14—H142	108.3	H211—C21—H213	107.7

### Hydrogen-bond geometry (Å, °)

**Table:**

*D*—H···*A*	*D*—H	H···*A*	*D*···*A*	*D*—H···*A*
C10—H102···N7^i^	0.98	2.67	3.468 (2)	139.
C3—H31···O1^ii^	0.94	2.67	3.569 (5)	159.

Symmetry codes: (i) −*x*, −*y*+2, −*z*+1; (ii) −*x*+1, −*y*+1, −*z*+1.
